# Glucose transporter-2 regulation of VMN GABA neuron metabolic sensor and transmitter gene expression

**DOI:** 10.1038/s41598-024-64708-y

**Published:** 2024-06-20

**Authors:** Sagor C. Roy, Subash Sapkota, Madhu Babu Pasula, Sushma Katakam, Rami Shrestha, Karen P. Briski

**Affiliations:** School of Basic Pharmaceutical and Toxicological Sciences, College of Pharmacy, University of Louisiana at Monroe, Rm 356 Bienville Building, 1800 Bienville Drive, Monroe, LA 71201 USA

**Keywords:** GLUT2 siRNA, GABA neuron, GAD1/2, nNOS, AMPK, Glucagon, Gene expression analysis, Neurology

## Abstract

Glucose transporter-2 (GLUT2) monitors cellular glucose uptake. Astrocyte GLUT2 controls glucose counterregulatory hormone secretion. In vivo gene silencing and laser-catapult-microdissection tools were used here to investigate whether ventromedial hypothalamic nucleus (VMN) GLUT2 may regulate dorsomedial (VMNdm) and/or ventrolateral (VMNvl) γ-aminobutyric acid (GABA) neurotransmission to control this endocrine outflow in female rats. VMN GLUT2 gene knockdown suppressed or stimulated hypoglycemia-associated glutamate decarboxylase (GAD)1 and GAD2 mRNA expression in VMNdm versus VMNvl GABAergic neurons, respectively. GLUT2 siRNA pretreatment also modified co-expressed transmitter marker gene profiles in each cell population. VMNdm GABA neurons exhibited GLUT2 knockdown-sensitive up-regulated 5’-AMP-activated protein kinase-alpha1 (AMPKα1) and -alpha2 (AMPKα2) transcripts during hypoglycemia. Hypoglycemic augmentation of VMNvl GABA neuron AMPKα2 was refractory to GLUT2 siRNA. GLUT2 siRNA blunted (VMNdm) or exacerbated (VMNvl) hypoglycemic stimulation of GABAergic neuron steroidogenic factor-1 (SF-1) mRNA. Results infer that VMNdm and VMNvl GABA neurons may exhibit divergent, GLUT2-dependent GABA neurotransmission patterns in the hypoglycemic female rat. Data also document differential GLUT2 regulation of VMNdm versus VMNvl GABA nerve cell SF-1 gene expression. Evidence for intensification of hypoglycemic hypercorticosteronemia and -glucagonemia by GLUT2 siRNA infers that VMN GLUT2 function imposes an inhibitory tone on these hormone profiles in this sex.

## Introduction

Glucose is the primary energy fuel used by the brain, where it is consumed at a disproportionately high rate compared to other organs. Hypoglycemia can hamper high energy-requiring neuron functions, including maintenance of transmembrane electrolyte gradients and synaptic transmission, thereby causing neurological impairment or injury to vulnerable nerve cell populations. Sensory cues on brain cell metabolic stability originate in a small number of neural structures, and are provided to the CNS glucostatic regulatory network to shape autonomic, neuroendocrine, and behavioral outflow that maintains blood glycemic profiles within an optimal physiological range^1^. Multiple sensory mechanisms operate in the brain, including screening of glucose at the critical junctures of (1) cellular uptake involving plasma membrane transport^2,3^ and (2) phosphorylation-mediated commitment to glycolytic processing^4^. Unlike other integral facilitative plasma membrane glucose transporters (GLUT), GLUT2 exhibits a relatively low affinity for glucose which likely enables its impediment-free transport over a wide physiological concentration range^5–7^. Brain astrocytes are essential contributors to neuro-metabolic stability; as a principal component of the blood–brain barrier, this glial cell type functions as the primary site of glucose acquisition by the brain^8–11^. In the astrocyte cell compartment, glucose is assembled into the energy reserve glycogen or converted to the trafficable monocarboxylate L-lactate to fuel nerve cell mitochondrial energy production^12–16^. Astrocyte GLUT2 activity is reportedly crucial for optimal counterregulation during pharmacological neuro-glucopenia^17^, but the neuroanatomical location(s) in which this sensor exerts control of physiological counterregulatory hormone secretion patterns have not been identified.

An expansive, dedicated glucose-regulatory network in the brain defends glucose homeostasis; the hypothalamus, the hierarchal autonomic motor center in the brain, operates within this circuitry to impose final, coordinated control of neural outflow that subserves that function^1^. Neurons in the ventromedial hypothalamic nucleus (VMN), a prominent component of the mediobasal hypothalamus, express gene transcripts that encode protein markers for neurochemicals that affect glucagon and corticosterone secretion, namely the amino acid transmitters γ-aminobutyric acid (GABA) and glutamate, the labile lipid-permeable gas nitric oxide (NO), and the neuropeptide growth hormone-releasing hormone (Ghrh)^18,19^. The high-sensitivity energy gauge 5’-AMP-activated protein kinase (AMPK) undergoes activation via phosphorylation in response to increases in the cellular AMP/ATP ratio^20–22^. Ventromedial hypothalamic AMPK is implicated in neural regulation of hypoglycemic patterns of counter regulatory hormone outflow^23,24^. VMN GABAergic neurons express AMPK alpha subunit mRNAs and gene product proteins, and show up-regulated AMPK alpha phosphorylation during hypoglycemia^25–28^. VMN GABAergic and nitrergic neurons evidently participate in metabolic coupling with astrocytes as transmitter marker protein profiles in each nerve cell population are sensitive to astrocyte-derived lactate signal volume^28,29^.

Hypothalamic astrocyte primary cultures express GLUT2, which regulates glucose and glycogen metabolism in those cells^30^. Current research applied gene knockdown tools in an in vivo experimental framework incorporating a validated model for insulin-induced hypoglycemia (IIH) to examine the hypothesis that VMN GLUT2 governs eu- and/or hypoglycemic patterns of counterregulatory hormone secretion. In our laboratory, proof of concept of astrocyte lactate involvement in glucose-regulatory transmitter signaling and counter-regulatory hormone secretion^28,29,31^ was acquired using a characterized ovariectomized (OVX) female rat model involving re-establishment of circulating estradiol levels at physiological, estrous cycle-like concentrations^32^. Here, a similar model was pretreated by bilateral administration of self-delivering Accell™ GLUT2 or control/scramble siRNA to the VMN prior to subcutaneous (*sc*) insulin (INS) or vehicle injection. Combinatory immunocytochemistry/laser-catapult-microdissection and single-cell multiplex RT-PCR methods were used to investigate the corollary premise that VMN GABAergic neuron function may be subject to GLUT2 control. Individual neurons identified in situ for glutamate decarboxylase_65/67_ (GAD _65/67_)-immunoreactivity were collected separately from the two prominent neuroanatomical divisions of the VMN, i.e. dorsomedial VMN (VMNdm) and ventrolateral VMN (VMNvl), to determine if one or both GABAergic nerve cell subpopulations exhibit transcriptional reactivity to VMN GLUT2 gene silencing. Individual VMNdm or VMNvl GABA nerve cell lysates were analyzed for effects of GLUT2 gene silencing alone or tandem GLUT siRNA/INS treatment on GAD1/GAD_67_ and GAD2/GAD_65_ mRNA expression, as well as PRKAA1/5’-AMP-activated protein kinase-alpha1 (AMPKα1) and PRKAA2/AMPKα2 transcript profiles. In light of evidence for neuronal co-expression of GABA and glutamate^33^, the prospect that VMNdm and/or VMNvl GAD_65/67_-immunopositive neurons may express GLUT2-sensitive markers for additional counterregulatory neurotransmitters was addressed here by expansion of multiplex gene profile analysis to include measurement of neuronal nitric oxide synthase (nNOS), vesicular glutamate transporter-1 (GLT1), and Ghrh mRNAs.

The transcription factor Nr5a1/steroidogenic factor-1 (SF-1) is uniquely expressed in the VMN, where it has been localized exclusively to the VMNdm^34,35^. Normal development of VMN cytoarchitecture and nerve cell phenotypic traits involves SF-1^36–40^. SF-1 function is crucial for neural regulation of systemic energy and glucose homeostasis^41–46^. Here, VMNdm and VMNvl GABAergic nerve cell lysates were analyzed for SF-1 mRNA expression to confirm whether these transcripts are only expressed in VMNdm, and to determine if this gene profile is responsive to GLUT2.

## Materials and methods

### Animals

Adult female Sprague Dawley adult rats (240–290 gm *bw*; 3 months of age) were housed in groups (2–3 animals per cage) under a 14 h light/10 h dark cycle (lights on at 05.00 h). Animals had unrestricted access to standard laboratory chow (Harlan Teklad LM-485; Harlan Industries, Madison, WI) and water, and were accustomed to daily handling. Study protocols and procedures were carried out in conformity with the NIH Guide for Care and Use of Laboratory Animals, 8^th^ Edition, with ULM Institutional Animal Care and Use Committee approval. All studies were performed in accord with ARRIVE guidelines.

### Experimental design

Animals were randomly assigned to one of four treatment groups (*n* = 6/group). On day 1, rats were anesthetized by intraperitoneal injection of ketamine/xylazine (0.1 mL/100 g *bw*; 90 mg ketamine:10 mg xylazine/mL; Henry Schein Animal Health, Dublin, OH), then bilaterally ovariectomized and implanted with a subcutaneous (*sc*) capsule filled with 17β estradiol-3-benzoate (30 µg/mL safflower oil; 10 mm/100 g *bw*; 0.062 in. *i.d*., 0.125 in. *o.d*.). In the current study design, circulating plasma estradiol concentrations in female subjects were standardized to minimize variability caused by dissimilar patterns of endogenous estradiol secretion at distinct estrous cycle stages. Mean plasma estradiol levels resulting from this replacement protocol (i.e. 22 pg/ml^32^) approximate concentrations measured during metestrus stage in 4-day cycling ovary-intact rats adult rat^47^. Anesthetized animals were also bilaterally injected into the VMN with Scramble siRNA (SCR; groups 1 and 3; Accell™ Control Pool Non-Targeting, 500 pmol; prod. no. D-001910-10-20; Horizon Discovery, Waterbeach, UK Dharmacon™, UK) or GLUT2 siRNA (groups 2 and 4; Accell™ Rat GLUT2 siRNA, set of 4, 500 pmol; prod. no. EQ-099803-00-0010; Horizon Disc.), aided by motorized computer-controlled Neurostar stereotactic drill and injection robot, as described^28^. Our studies show that this knockdown reagent causes significant down-regulation of GLUT2 protein expression in primary hypothalamic astrocytes cultures in vitro^48,49^. After surgery, animals were injected with ketoprofen (1 mg/ml/kg *bw sc*; Bayer Health Care LLC, Animal Health Division, S. Mission, Kansas) and enrofloxacin (10 mg/0.1 mL IM; Bayer Health), and treated by topical application of 0.25% bupivacaine (Hospira Inc., IL) to closed incisions. After full recovery from surgery, animals were transferred to individual cages. At 09:00 h on day 7, animals were injected *sc* with vehicle (V; sterile insulin diluent; Eli Lilly & Company, Indianapolis, IN; groups 1 and 2;) or neutral protamine Hagedorn insulin (INS; 10 U/kg *bw*^50^; Eli Lilly; groups 3 and 4). Unanesthetized animals were sacrificed at 10:00 h by rapid decapitation in a rodent guillotine for brain and trunk blood collection. Individual dissected brains were snap-frozen in dry ice-cooled isopentane followed by storage at -80 °C. Plasma was stored at -20 °C.

### Laser-catapult-microdissection of VMNdm and VMNvl GABAergic neurons and astrocytes

Consecutive 10 μm-thick frozen sections were cut through the VMN of each brain between −1.8 mm to −2.3 mm posterior to *bregma* for immunohistochemistry and laser-catapult-microdissection. Individual 10 μm-thick fresh frozen sections were mounted on polyethylene naphthalate membrane-coated slides (prod. no. 415190-9041-000; Carl Zeiss Microscopy LLC, White Plains, NY). Immunolabeling of GABA nerve cells was performed as described^18,28,29,51–54^. Tissues were fixed (5 min) with ice-cold acetone, then blocked (1 h) with 1.5% normal goat serum (prod. no. S-2000, Vector Laboratories, Burlingame, CA) in Tris-buffered saline, pH 7.4, (TBS), 0.05% Triton X-100, before incubation (36–48 h; 4 °C) with a primary antiserum raised in rabbit against GAD_65/67_ (prod. no. ABN904, 1:1000; Millipore Sigma, Burlington, MA). Sections were then exposed (1 h) to a horseradish peroxidase-labeled goat anti-rabbit secondary antiserum (prod. no. PI-1000, 1:1000; Vector Lab.). VMNdm and VMNvl astrocytes were identified in situ by immunostaining for the marker protein glial fibrillary acidic protein (GFAP) in preparation for laser-catapult-microdissection for the purpose of validating efficacy of GLUT2 gene knockdown in each location. Briefly, acetone-fixed sections were blocked (1 h) with 1.5% normal horse serum (prod. no. S-2000, Vector Lab.) in TBS containing 0.05% Triton X-100, then incubated (36–48 h; 4 °C) with a mouse mouse monoclonal antibody against GFAP (prod. no. 3670S, 1:500; Cell Signaling Technology, Inc., Danvers, MA). Sections were then sequentially incubated (2 h) with a biotinylated horse anti-mouse secondary antibody (prod. no. BA-2000, 1:1000, Vector Lab.) and ABC reagent (1 h) (prod. no. PK-7100, Vector Lab.). GAD_65/67_–immunoreactive (-ir) neurons and GFAF-ir astrocytes were visualized using Vector ImmPACT 3,3-diaminobenzidine peroxidase substrate kit reagents (prod. no. SK-4105; Vector Lab.), as reported^29,31,53,54^. Individual VMNdm and VMNvl GAD_65/67_–ir neurons and GFAP-ir astrocytes were removed from tissue sections using a Zeiss P.A.L.M. UV-A microlaser IV (Carl Zeiss), and collected into an adhesive cap (prod. no. 415190-9181-000; Carl Zeiss) containing SingleShot Cell Lysis kit buffer (prod. no. 1725080; Bio-Rad Laboratories, Hercules, CA).

### Single-cell multiplex reversed transcription quantitative polymerase chain reaction (RT-qPCR) analysis

*Complementary DNA (cDNA) Synthesis and Amplification*: Individual neurons collected into lysis buffer were sequentially incubated at 25 °C (10 min), then 75 °C (5 min) in a Bio-Rad iCyclerQ RT-PCR Detection System. RNA samples were reverse-transcribed to cDNA using iScript™ Advanced cDNA Synthesis kit reagents (prod. no. 1725038; Bio-Rad), using described methods^19,27,55^. Preamplification master mix was prepared by combining Bio-Rad PrimePCR™ PreAmps for SYBR® Green Assays for GAD1/GAD67 (prod. no. qRnoCID0004554), GAD2/GAD65 (prod. no. qRnoCID0003485), NOS1/nNOS (prod. no. qRnoCED0009301), Ghrh (prod. no. qRnoCID0007723), Slc1A2/GLT-1 (prod. no. qRnoCED0005967), PRKAA1 (prod. no. qRnoCID0001262), PRKAA2 (prod. no. qRnoCID0006799), Nr5a1/SF-1 (prod. no. qRnoCID0001458), Slc2A2 (prod. no. qRnoCID0009479), and GAPDH (prod. no. qRnoCID0057018) with SsoAdvanced™ PreAmp Supermix (prod. no. 1725160 Bio-Rad**).** Pre-amplification master mix (9.5 µL) was added to each cDNA sample prior to initial incubation at 95 °C (3 min), followed by 22 cycles of sequential 95 °C (15 s) and 60 °C (4 min) incubations. *RT-qPCR Analysis:* Pre-amplified cDNA samples were diluted by addition IDTE (prod. no. 11-05-01-05; 1X TE solution; Integrated DNA Technologies, Inc., Coralville, IA), and combined with Bio-Rad primers [GAD1/GAD_67_ (prod. no. qRnoCID0004554), GAD2/GAD_65_ (prod. no. qRnoCID0003485), NOS1/nNOS (prod. No. qRnoCED0009301), Ghrh (prod. no. qRnoCID0007723), Slc1A2/GLT-1 (prod. no. qRnoCED0005967), PRKAA1 (prod. no. qRnoCID0001262), PRKAA2 (prod. no. qRnoCID0006799), Nr5a1/SF-1 (prod. no. qRnoCID0001458), Slc2A2 (prod. no. qRnoCID0009479), and GAPDH (prod. no. qRnoCID0057018)] and iTaq™ Universal SYBR® Green supermix (prod. no. 1725121*;* Bio-Rad). Hard-Shell 384-Well PCR plates (prod. no. HSP3805, Bio-Rad) were used to run PCR reactions in a Bio-Rad CFX384™ Touch Real-Time PCR Detection System under the following conditions: denaturation at 95 °C (30 s), followed by 40 cycles of initial 3 s 95 °C incubation, then secondary 45 s incubation at 60.5 °C for PRKAA1 and PRKAA2, 60 °C for GAD1/GAD_67_and GAD2/GAD_65_, 59.1 °C for Nr5a1/SF-1, 58.0 °C for Ghrh and NOS1/nNOS, or 57.3 °C for Slc1A2/GLT-1, Slc2A2, and GAPDH. Melt curve analyses were carried out to identify nonspecific products and primer dimers. Data were analyzed by the 2^−ΔΔCt^ or comparative Ct method^56^.

### Glucose and counter-regulatory hormone measurements

Plasma glucose levels were measured using an ACCU-CHECK Aviva plus glucometer (Roche Diagnostic Corporation, Indianapolis, IN)^50^. Plasma corticosterone and glucagon concentrations were analyzed with commercial kit reagents (Corticosterone ELISA kit, prod. no. ADI-900-097; Enzo Life Sciences, Inc., Farmingdale, NY; Glucagon ELISA kit, prod. no. EZGLU-30K, EMD Millipore, Billerica, MA).

### Statistics

Mean normalized VMNdm or VMNvl mRNA, glucose, and counterregulatory hormone values were analyzed between treatment groups by two-way analysis of variance and Student Newman Keuls *post-hoc* test. Differences of *p* < 0.05 were considered significant. In each figure, statistical differences between specific pairs of treatment groups are denoted as follows: *p < 0.05; **p < 0.01; ***p < 0.001; ****p < 0.0001.

### Ethical approval

Studies performed here were approved by the University of Louisiana Monroe Institutional Animal Care and Use Committee, reference no. 19AUG-KPB-01, in accordance with the National Institutes of Health (NIH) Guide for Care and Use of Laboratory Animals, 8th Edition.

## Results

Astrocyte GLUT2 acts within the brain to shape glucose counterregulatory outflow. VMN astrocytes engage in metabolic coupling with neurons that function within the glucostatic network, including counterregulation-inhibiting GABAergic nerve cells. Current research employed in vivo gene knockdown tools in the context of a validated experimental model for IIH to investigate whether VMN GLUT2 controls patterns of counterregulatory hormone secretion in the female rat during eu- and/or hypoglycemia. Combinatory immunocytochemistry/laser-catapult-microdissection/multiplex single-cell qPCR methods were also used to determine if VMNdm and/or VMNvl GABA neurons may be targets of GLUT2 regulatory action in this sex.

**Figure 1:**
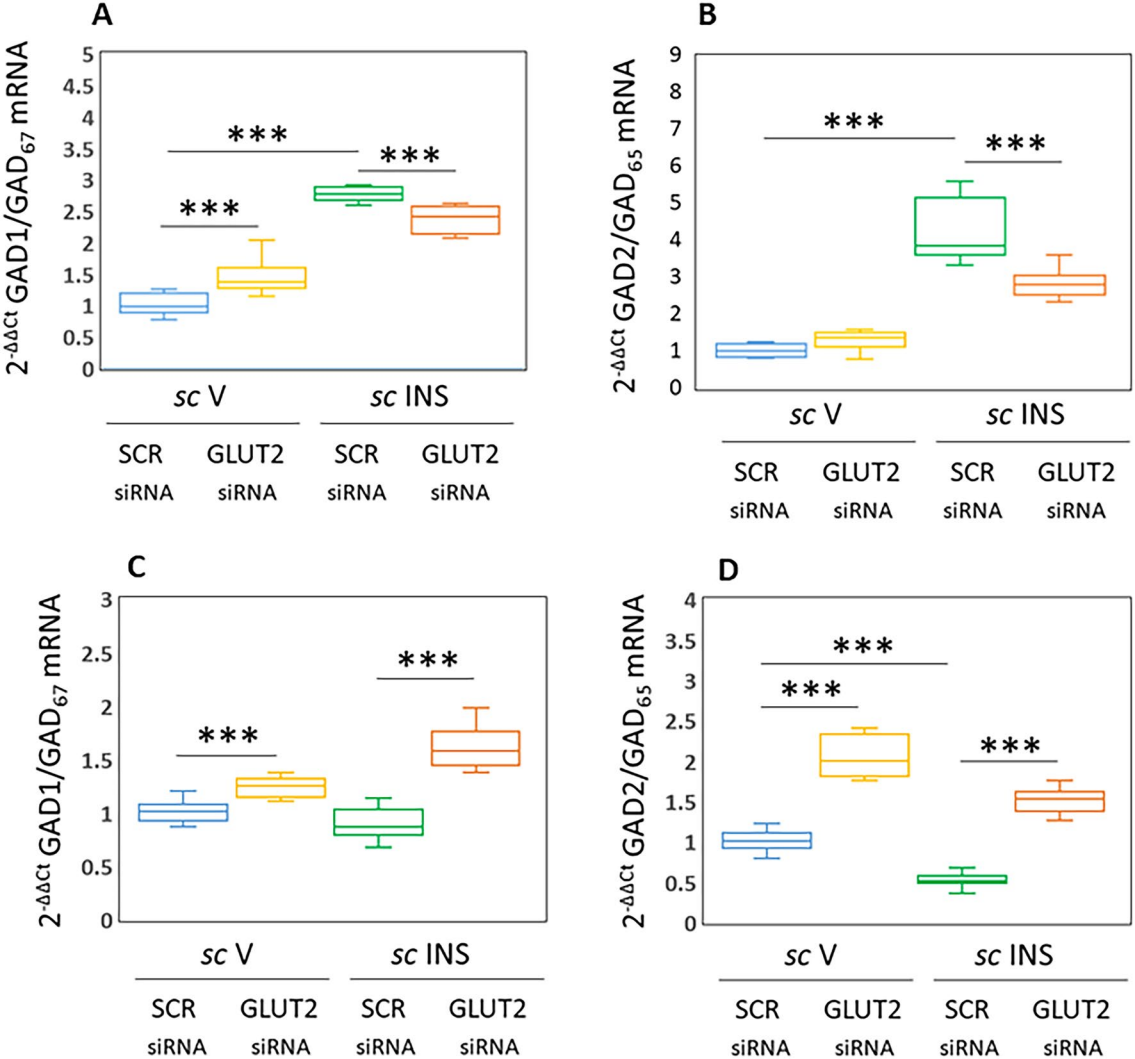
Effects of Ventromedial Hypothalamic Nucleus (VMN) Glucose Transporter-2 (GLUT) Gene Silencing on Dorsomedial VMN (VMNdm) or Ventrolateral VMN (VMNvl) γ-Aminobutyric Acid (GABA) Neuron Glutamate Decarboxylase-1 (GAD1/GAD67) and GAD2/GAD Gene Expression in the Female Rat. Groups of adult ovariectomized, estradiol-implanted female rats (*n* = 6/group) were pretreated by bilateral stereotactic administration of control/scramble (SCR) or GLUT2 siRNA to the VMN, then injected subcutaneously (*sc*) with vehicle (V) or neutral protamine Hagedorn insulin (INS; 10.0 U/kg *bw*) one hour before sacrifice. Individual VMNdm and VMNvl GABAergic neurons were labeled in situ by immunocytochemical methods for GAD65/67-immunoreactivity (-ir) prior to laser-catapult-microdissection for single-cell multiplex qPCR analysis. mRNA data were normalized to the housekeeping gene GAPDH by the 2^-ΔΔCt^ method. Data are presented here in box-and-whisker plot format, which displays the median, lower and upper quartiles, and lower and upper extremes of a data set. Plots depict normalized GAD1/GAD_67_ [**A** (VMNdm) and **C** (VMNvl)] or GAD2/GAD_65_ mRNA [**B** (VMNdm) and **D** (VMNvl) values for GABAergic neurons collected from the following treatment groups: SCR siRNA/*sc* V (blue box-and-whisker plots, *n* = 12), GLUT2 siRNA/*sc* V (yellow box-and-whisker plots; *n* = 12), SCR siRNA/ *sc* INS (green box-and-whisker plots; *n* = 12), GLUT2 siRNA/*sc* INS (orange box-and-whisker plots; *n* = 12). Statistical differences between discrete pairs of treatment groups are denoted as follows: *p < 0.05; **p < 0.01; ***p < 0.001. (**A**) VMNdm GAD1/GAD_67_. (**B**) VMNdm GAD2/GAD_65_. (**C**) VMNvl GAD1/GAD_67_. (**D**) VMNvl GAD2/GAD_65_.

Figure 1 depicts effects of bilateral SCR versus GLUT2 siRNA administration to the VMN on GABAergic neuron GAD1 [Figs. 1A (VMNdm) and C (VMNvl)] and GAD2 [Figs. 1B (VMNdm) and D (VMNvl)] mRNA levels. Data show that GLUT2 gene silencing increased GABA nerve cell GAD1 [Fig. 1A; SCR siRNA/*sc* V (blue box-and-whisker plot) versus GLUT2 siRNA/*sc* V (yellow box-and-whisker plot); F_(3,44)_: 110.72, *p* < 0.001, Knockdown main effect: F_(1,44)_: 014; *p* = 0.708; INS main effect: F_(1,44)_: 297.27; *p* < 0.001, Knockdown/INS interaction: F_(1,44)_: 34.75; *p* < 0.001], but not GAD2 [Fig. 1B; F_(3,44)_: 120.14, *p* < 0.001, Knockdown main effect: F_(1,44)_: 17.08; *p* < 0.0001; INS main effect: F_(1,44)_: 305.21; *p* < 0.001, Knockdown/INS interaction: F_(1,44)_: 38.12; *p* < 0.001] gene expression. IIH up-regulated both GAD mRNAs in VMNdm GABA nerve cells [SCR siRNA/*sc* INS (green box-and-whisker plot) versus GLUT2 siRNA/*sc* INS (orange box-and-whisker plot); these stimulatory transcriptional responses were attenuated by GLUT2 siRNA pretreatment.

Figures 1C,D illustrate VMNvl GABAergic nerve cell GAD1 and GAD2 gene expression profiles in SCR versus GLUT2 siRNA-pretreated female rats. Results show that GLUT2 gene knockdown increased both GAD1 [F_(3,44)_: 59.59, *p* < 0.001, Knockdown main effect: F_(1,44)_: 134.63; *p* < 0.0001; INS main effect: F_(1,44)_: 9.99; *p* = 0.003, Knockdown/INS interaction: F_(1,44)_: 34.00, *p* < 0.001] and GAD2 gene expression [F_(3,44)_: 186.90, *p* < 0.001, Knockdown main effect: F_(1,44)_: 445.56; *p* < 0.0001; INS main effect: F_(1,44)_: 114.92; *p* < 0.001, Knockdown/INS interaction: F_(1,44)_: 0.214; *p* = 0.646] in VMNvl GABA neurons. GAD1 mRNA levels in this cell population were unaffected by hypoglycemia, whereas GAD2 gene expression was inhibited. Both GAD variant gene profiles were significantly higher in VMNvl GABAergic nerve cells collected from GLUT2 siRNA/*sc* INS versus SCR siRNA/*sc* INS treatment groups. Collectively, results presented in Fig. 1 show that GLUT2 is inhibitory to baseline GABA nerve cell GAD1 gene expression in both the VMNdm and VMNvl, but only regulates basal GAD2 transcription in the latter subdivision. Data also disclose distinctive, GLUT2-dependent division-specific GAD isoform gene responses to hypoglycemia, e.g. up- versus down-regulation in VMNdm versus VMNvl.

**Figure 2:**
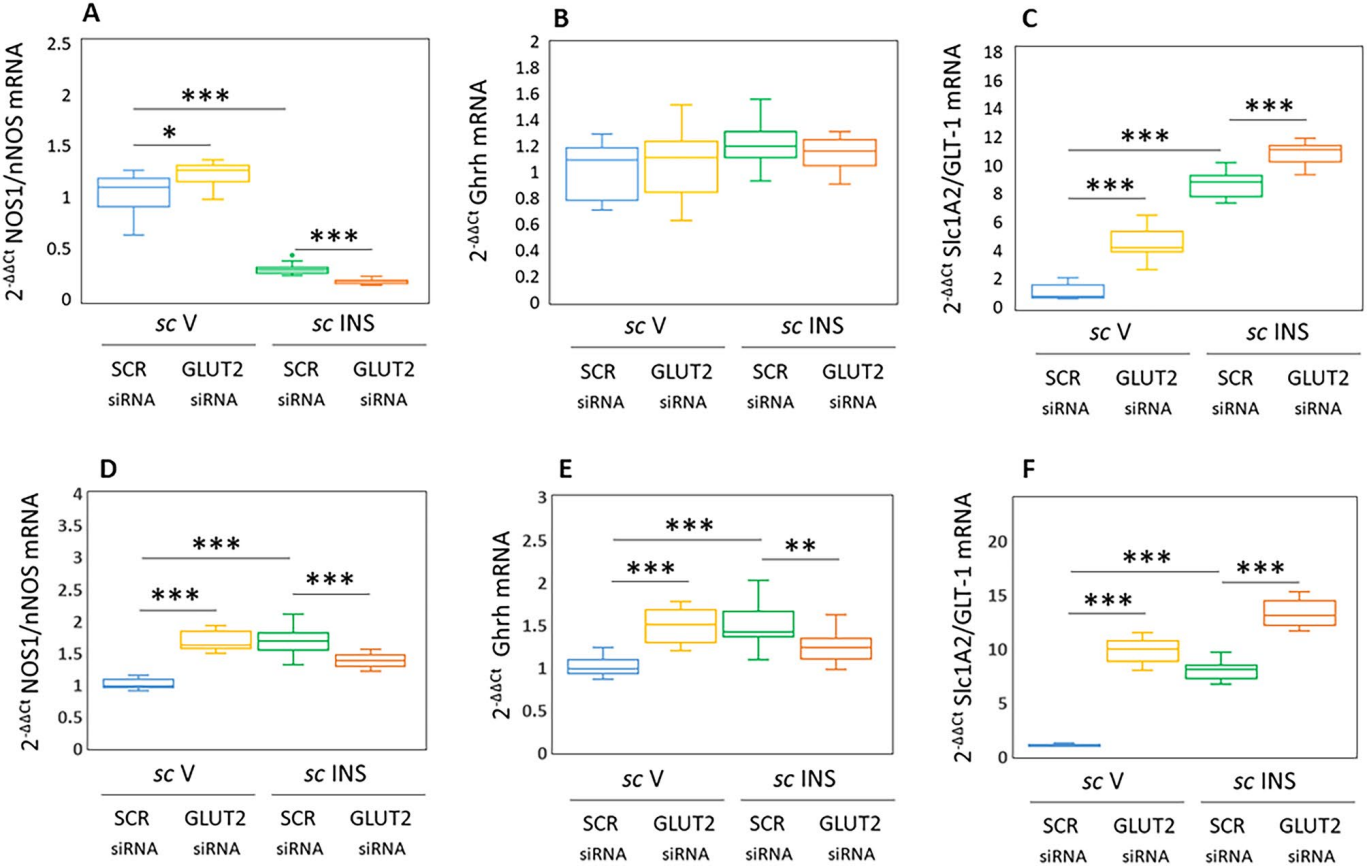
VMNdm versus VMNvl GABAergic Nerve Cell Co-Expressed Neurotransmitter Marker Gene Profiles: Impact of VMN GLUT2 Gene Knockdown. Box-and-whisker plots depict normalized values for GABAergic nerve cell mRNAs encoding neuronal nitric oxide synthase (NOS1/nNOS; marker for nitric oxide), growth hormone-releasing hormone (Ghrh), or vesicular glutamate transporter-1 (GLT-2; marker for glutamate). Plots depict normalized NOS1/nNOS [**A** (VMNdm) and **D** (VMNvl)], Ghrh [**B** (VMNdm) and **E** (VMNvl)], or GLT-1 [**C** (VMNdm) and **F** (VMNvl)] values for VMNdm or VMNvl GABA neurons collected from SCR siRNA/*sc* V (blue box-and-whisker plots, *n* = 12), GLUT2 siRNA/*sc* V (yellow box-and-whisker plots; n = 12), SCR siRNA/ *sc* INS (green box-and-whisker plots; *n* = 12), or GLUT2 siRNA/*sc* INS (orange box-and-whisker plots; *n* = 12) treatment groups. Statistical differences between discrete pairs of treatment groups are indicated by *p < 0.05; **p < 0.01; or ***p < 0.001. (**A**) VMNdm NOS1/nNOS. (**B**) VMNdm Ghrh. (**C**) VMNdm GLT-1. (**D**) VMNvl NOS1/nNOS. (**E**) VMNvl Ghrh. (**F**) VMNvl GLT-1.

Figure 2 depicts effects of SCR versus GLUT2 siRNA on co-expressed counterregulatory neurochemical marker gene profiles, i.e. NOS1 (Fig. 2A and D), Ghrh (Fig. 2B and E), and GLT-1 (Fig. 2C and F). As shown in Fig. 2A and D, GLUT2 gene silencing up-regulated NOS1 mRNA levels in VMNdm [F_(3,44)_: 199.52, *p* < 0.001, Knockdown main effect: F_(1,44)_: 0.87; *p* = 0.357; INS main effect: F_(1,44)_: 578.98; *p* < 0.001, Knockdown/INS interaction: F_(1,44)_: 18.72; *p* < 0.001] and VMNvl [F_(3,44)_: 58.02, *p* < 0.001, Knockdown main effect: F_(1,44)_: 17.69; *p* < 0.0001; INS main effect: F_(1,44)_: 19.93; *p* < 0.001, Knockdown/INS interaction: F_(1,44)_: 136.43; *p* < 0.001] GABA neurons, respectively. Data show that IIH diminished NOS1 gene expression in VMNdm GABA nerve cells, and that this inhibitory transcriptional response was exacerbated by GLUT2 siRNA pretreatment. VMNvl GABA neurons exhibited amplified NOS1 transcript profiles in response to hypoglycemia, a stimulatory response that was blunted by GLUT2 gene knockdown. In VMNdm GABAergic neurons, Ghrh mRNA levels (Fig. 2B) were refractory to GLUT2 gene silencing and hypoglycemia F_(3,44)_: 1.94, *p* = 0.137, Knockdown main effect: F_(1,44)_: 0.022; *p* = 0.882; INS main effect: F_(1,44)_: 4.74; *p* = 0.035, Knockdown/INS interaction: F_(1,44)_: 1.06; *p* = 0.309]. In contrast, Ghrh mRNA levels in VMNvl GABA nerve cells (Fig. 2E) were enhanced by GLUT2 gene silencing, and hypoglycemic up-regulation of this gene profile was attenuated by GLUT2 siRNA pretreatment [F_(3,44)_: 17.89, *p* < 0.001, Knockdown main effect: F_(1,44)_: 4.28; *p* = 0.044; INS main effect: F_(1,44)_: 4.22; *p* = 0.046, Knockdown/INS interaction: F_(1,44)_: 4515; *p* < 0.001]. Gene transcripts encoding the glutamate transporter GLT-1, a marker for glutamate neurotransmission, were up-regulated by GLUT2 gene knockdown in GABA neurons acquired from either the VMNdm [Fig. 2C; F_(3,44)_: 303.21, *p* < 0.001, Knockdown main effect: F_(1,44)_: 123.91; *p* < 0.0001; INS main effect: F_(1,44)_: 779.71; *p* < 0.001, Knockdown/INS interaction: F_(1,44)_: 6.00; *p* = 0.018] or VMNvl [Fig. 2F; F_(3,44)_: 299.50, *p* < 0.001, Knockdown main effect: F_(1,44)_: 552.50; *p* < 0.0001; INS main effect: F_(1,44)_: 309.62; *p* < 0.001, Knockdown/INS interaction: F_(1,44)_: 36.36; *p* < 0.001]. IIH stimulated this gene profile in both VMN divisions; this positive response was amplified by GLUT2 siRNA pretreatment in both locations. Data in Fig. 2 indicate that GLUT2 has a negative regulatory effect on baseline GABA neuron nNOS and GLT-1 gene expression in each VMN subdivision, but suppresses basal Ghrh mRNA levels only in the VMNvl. Interesting, outcomes document a directional shift in GLUT2 control of nNOS and Ghrh transcript levels, e.g. from inhibitory-to-stimulatory, during hypoglycemia.

**Figure 3:**
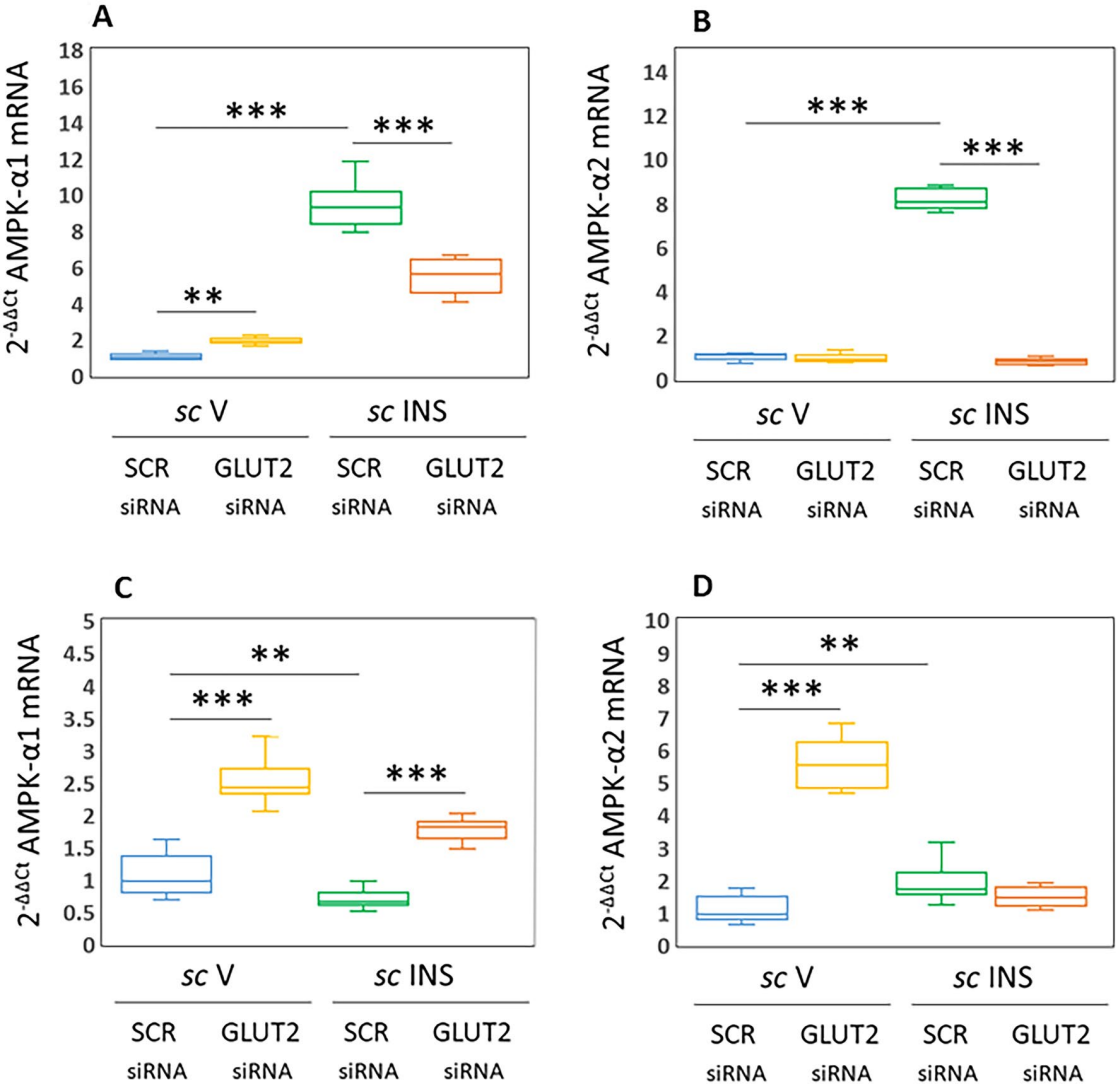
Effects of VMN GLUT2 siRNA Pretreatment on Hypoglycemic Patterns of PRKAA1/5’-AMP-Activated Protein Kinase-Alpha1 (AMPKα1) and PRKAA2/AMPKα2 Gene Expression in VMNdm versus VMNvl GABAergic Neurons. Plots depict normalized PRKAA1/AMPKα1 [**A** (VMNdm) and **C** (VMNvl)] or PRKAA2/AMPK α1 [**B** (VMNdm) and **D** (VMNvl)] mRNA measures for VMNdm or VMNvl GABA neurons collected from SCR siRNA/*sc* V (blue box-and-whisker plots, *n* = 12), GLUT2 siRNA/*sc* V (yellow box-and-whisker plots; *n* = 12), SCR siRNA/ *sc* INS (green box-and-whisker plots; *n* = 12), or GLUT2 siRNA/*sc* INS (orange box-and-whisker plots; *n* = 12) treatment groups. Statistical differences between discrete pairs of treatment groups are indicated by the following symbols: *p < 0.05; **p < 0.01; ***p < 0.001. (**A**) VMNdm PRKAA1/AMPKα1. (**B**) VMNdm PRKAA2/AMPKα2. (**C**) VMNvl PRKAA1/AMPKα1. (**D**) VMNvl PRKAA2/AMPKα2.

Figure 3 presents effects of GLUT2 gene knockdown on PRKAA1/AMPKα1 (Fig. 3A and C) and PRKAA2/AMPKα2 (Fig. 3B and D) mRNA levels in VMNdm versus VMNvl GABAergic neurons. Data show that GLUT2 gene silencing up-regulates PRKAA1 in both VMNdm [F_(3,44)_: 313.61, *p* < 0.001, Knockdown main effect: F_(1,44)_: 49.74; *p* < 0.0001; INS main effect: F_(1,44)_: 771.04; *p* < 0.001, Knockdown/INS interaction: F_(1,44)_: 120.05; *p* < 0.001] and VMNvl [F_(3,44)_: 99.25, *p* < 0.001, Knockdown main effect: F_(1,44)_: 245.55; *p* < 0.0001; INS main effect: F_(1,44)_: 46.17; *p* < 0.001, Knockdown/INS interaction: F_(1,44)_: 6.02; *p* = 0.018]. IIH increased PRKAA1 mRNA levels in VMNdm GABA neurons; GLUT2 siRNA pretreatment curbed this stimulatory transcriptional response. VMNvl GABA nerve cells showed down-regulated PRKAA1 gene expression during hypoglycemia; this response was reversed by GLUT2 gene knockdown. GLUT2 gene knockdown enhanced PRKAA2 mRNA levels in GABA neurons collected from VMNvl (Fig. 3D) [F_(3,44)_: 189.43, *p* < 0.001, Knockdown main effect: F_(1,44)_: 185.40; *p* < 0.0001; INS main effect: F_(1,44)_: 117.14; *p* < 0.001, Knockdown/INS interaction: F_(1,44)_: 265.76; *p* < 0.001], but not VMNdm (Fig. 3B) [F_(3,44)_: 1115.59, *p* < 0.001, Knockdown main effect: F_(1,44)_: 1172.752; *p* < 0.0001; INS main effect: F_(1,44)_: 1041.43; *p* < 0.001, Knockdown/INS interaction: F_(1,44)_: 1132.81; *p* < 0.001]. PRKAA2 gene expression was up-regulated by IIH in each location; GLUT2 siRNA pretreatment abolished this stimulatory response in the VMNdm, but not the VMNvl. Data in Fig. 3 show that GLUT2 inhibits baseline GABA neuron PRKAA1 and PRKAA2 gene expression according to VMN subdivision. Results provide unique evidence for divergent hypoglycemic effects on VMNdm versus VMNvl GABAergic nerve cell PRKAA1 mRNA, where GLUT2 corresponding stimulates or inhibits these gene profiles. Hypoglycemic up-regulation of PRKAA2 transcripts was GLUT2-dependent (VMNdm) or -independent (VMNvl).

**Figure 4:**
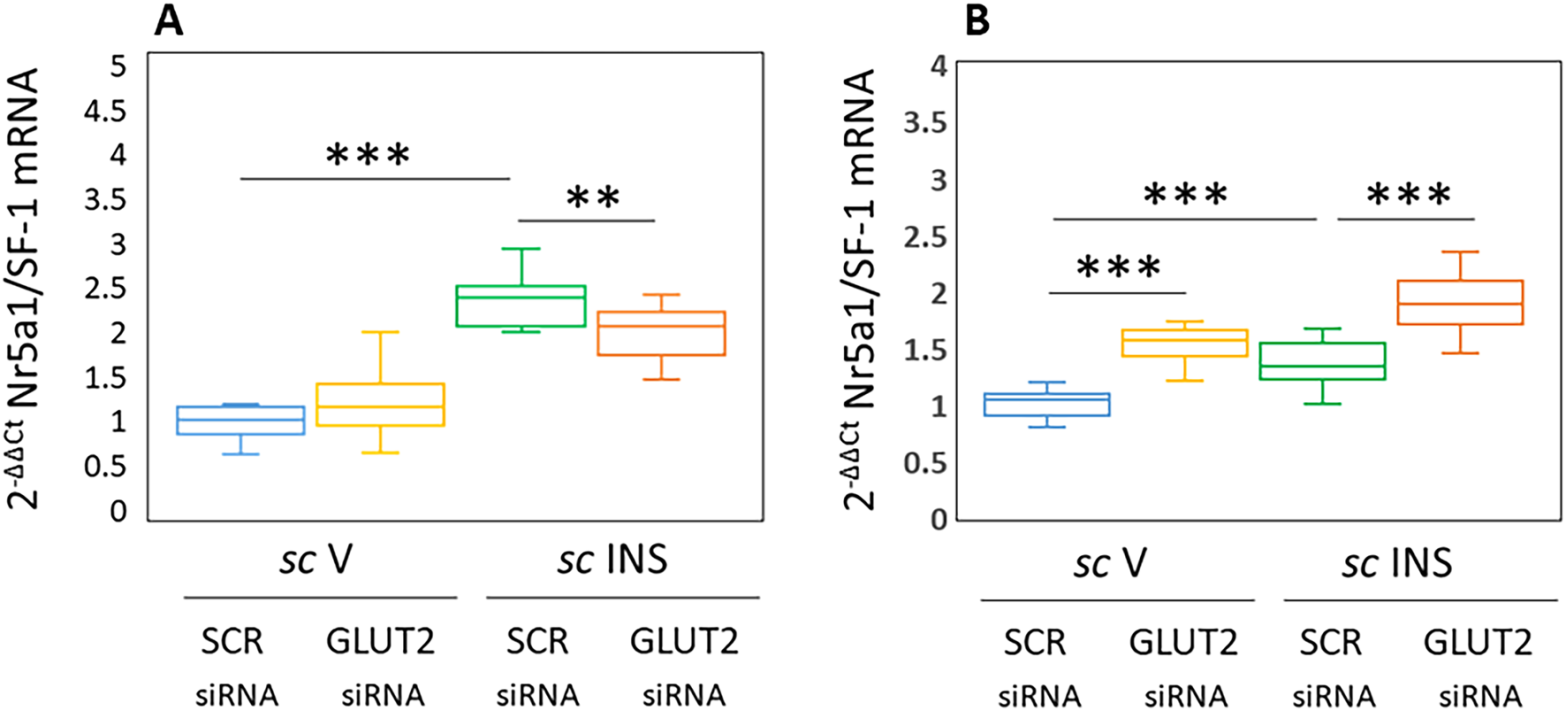
Eu- and Hypoglycemic Patterns of Nr5a1/Steroidogenic Factor-1 (SF-1) Gene Expression Profiles in VMNdm versus VMNvl GABAergic Neurons; Impact of VMN GLUT2 Gene Silencing. Plots depict normalized Nr5a1/SF-1 [**A** (VMNdm) and **B** (VMNvl)] mRNA measures for VMNdm or VMNvl GABA neurons collected from SCR siRNA/*sc* V (blue box-and-whisker plots, *n* = 12), GLUT2 siRNA/*sc* V (yellow box-and-whisker plots; *n* = 12), SCR siRNA/ *sc* INS (green box-and-whisker plots; *n* = 12), or GLUT2 siRNA/*sc* INS (orange box-and-whisker plots; *n* = 12) treatment groups. Statistical differences between discrete pairs of treatment groups are indicated by the following symbols: *p < 0.05; **p < 0.01; ***p < 0.001. (**A**) VMNdm Nr5a1/SF-1. (**B**) VMNvl Nr5a1/SF-1.

Figure 4 depicts effects of GLUT2 gene knockdown on VMNdm (Fig. 4A) versus VMNvl (Fig. 4B) GABA nerve cell SF-1 gene expression. Results indicate that GLUT2 siRNA up-regulates SF-1 mRNA levels in VMNvl [F_(3,44)_: 45.90, *p* < 0.001, Knockdown main effect: F_(1,44)_: 94.40; *p* < 0.0001; INS main effect: F_(1,44)_: 43.28; *p* < 0.001, Knockdown/INS interaction: F_(1,44)_: 0.01; *p* = 0.944], but not VMNdm [F_(3,44)_: 62.71, *p* < 0.001, Knockdown main effect: F_(1,44)_: 0.72; *p* = 0.402; INS main effect: F_(1,44)_: 175.45; *p* < 0.001, Knockdown/INS interaction: F_(1,44)_: 11.96; *p* = 0.001] GABA neurons. Hypoglycemia increased SF-1 gene expression in GABA nerve cells taken from each VMN division. GLUT2 siRNA pretreatment blunted or amplified this stimulatory response in the VMNdm and VMNvl, respectively. Outcomes here show that GLUT2 exerts inhibitory control of baseline SF-1 transcription in VMNvl, but not VMNdm GABA neurons. Data reveal hypoglycemia-associated up-regulation of this gene profile in each GABA cell population, responses that were stimulated (VMNdm) or inhibited (VMNvl) by GLUT2.

**Figure 5:**
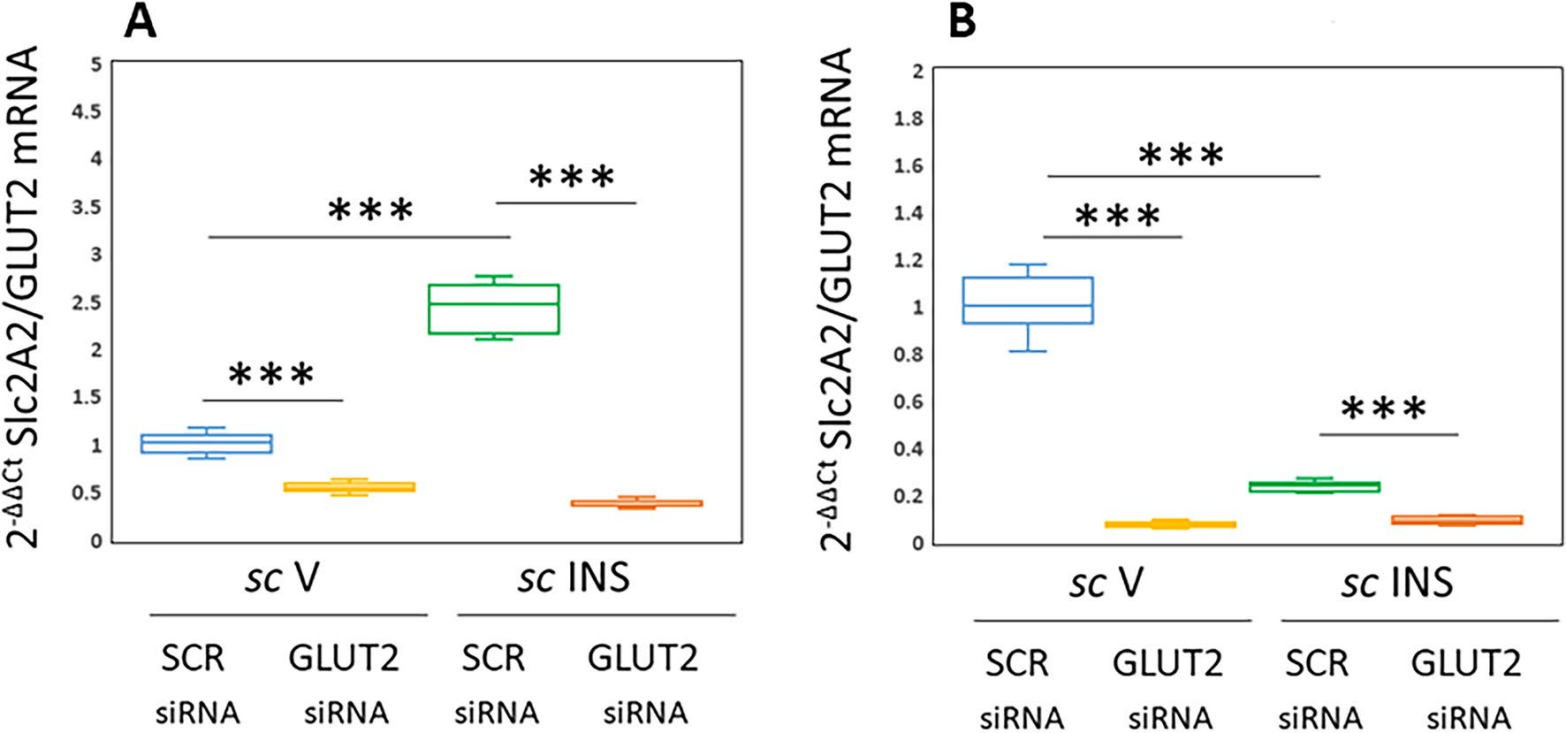
Efficacy of VMN GLUT2 Gene Knockdown on VMNdm versus VMNvl Astrocyte GLUT2 mRNA Expression. Individual VMNdm and VMNvl astrocytes identified in situ by immunocytochemical labeling for glial fibrillary acidic protein (GFAP)-ir were harvested by laser-catapult-microdissection for qPCR analysis of GLUT2 gene expression. Data were normalized to the housekeeping gene GAPDH by the 2^-ΔΔCt^ method, and presented in box-and-whisker plot format. Plots depict normalized GLUT2 [**A** (VMNdm) and **B** (VMNvl)] mRNA measures for VMNdm or VMNvl astrocytes acquired from the following treatment groups: SCR siRNA/*sc* V (blue box-and-whisker plots, *n* = 12), GLUT2 siRNA/*sc* V (yellow box-and-whisker plots; *n* = 12), SCR siRNA/ *sc* INS (green box-and-whisker plots; *n* = 12), GLUT2 siRNA/*sc* INS (orange box-and-whisker plots; *n* = 12). Statistical differences between discrete pairs of treatment groups are indicated by the following symbols: *p < 0.05; **p < 0.01; ***p < 0.001. (**A**) VMNdm Astrocyte GLUT2. (**B**) VMNvl Astrocyte GLUT2.

Data in Fig. 5 depicts effects of SCR or GLUT2 siRNA administration to the VMN on VMNdm (Fig. 5A) and VMNvl (Fig. 5B) astrocyte GLUT2 gene expression. Results indicate that GLUT2 gene silencing significantly decreased GLUT2 transcript profiles in astrocytes collected from the VMNdm [F_(3,44)_: 556.54, *p* < 0.001, Knockdown main effect: F_(1,44)_: 1013.93; *p* < 0.0001; INS main effect: F_(1,44)_: 252.10; *p* < 0.001, Knockdown/INS interaction: F_(1,44)_: 403.59; *p* < 0.0001] or VMNvl [F_(3,44)_: 623.53, *p* < 0.001, Knockdown main effect: F_(1,44)_: 922.85; *p* < 0.0001; INS main effect: F_(1,44)_: 455.75; *p* < 0.001, Knockdown/INS interaction: F_(1,44)_: 491.99; *p* < 0.0001]. Hypoglycemia caused opposite adjustments in astrocyte GLUT2 gene expression, as mRNA levels were increased in the VMNdm or decreased in the VMNvl. GLUT2 siRNA pretreatment diminished hypoglycemic patterns of GLUT2 gene expression in VMNdm and VMNvl astrocytes. Results provide evidence for efficacy of the current siRNA treatment paradigm for inhibition of astrocyte GLUT2 gene expression in both VMNdm and VMNvl.

**Figure 6:**
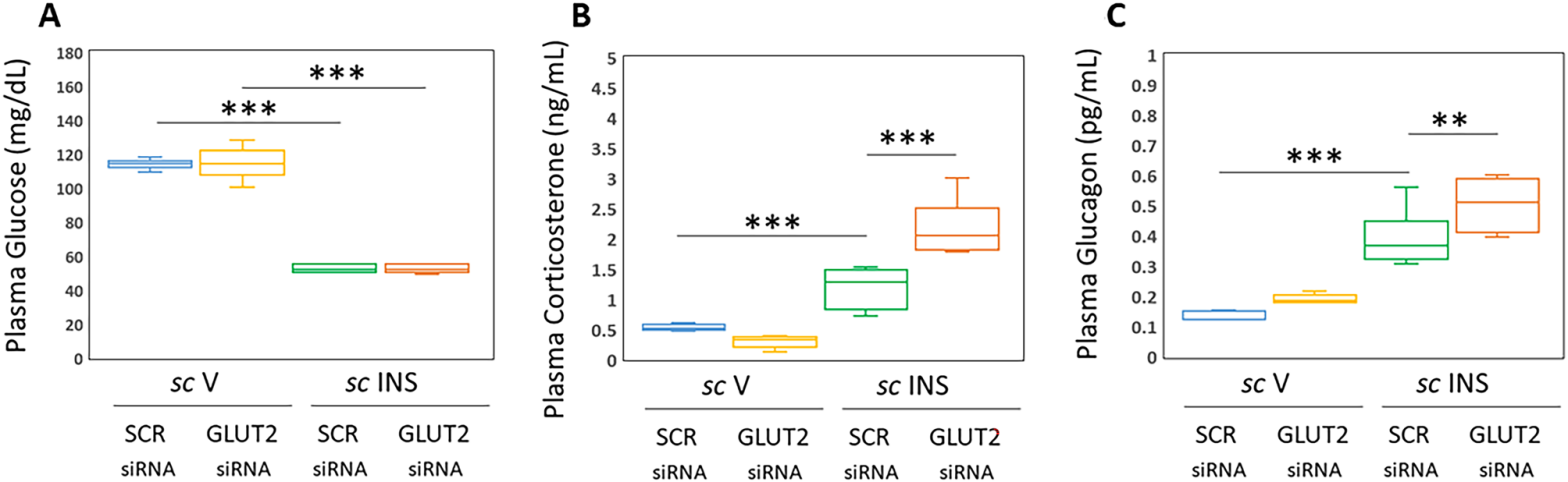
Effects of VMN GLUT2 Gene Knockdown on Plasma Glucose and Counterregulatory Hormone Profiles in the Adult Female Rat. Box-and-Whisker plots depict circulating glucose (**A**), corticosterone (**B**), or glucagon (**C**) concentrations for the following treatment groups: SCR siRNA/*sc* V (blue box-and-whisker plots, *n* = 6), GLUT2 siRNA/*sc* V (yellow box-and-whisker plots; *n* = 6), SCR siRNA/ *sc* INS (green box-and-whisker plots; *n* = 6), or GLUT2 siRNA/*sc* INS (orange box-and-whisker plots; *n* = 6) treatment groups. Statistical differences between discrete pairs of treatment groups are indicated by the following symbols: *p < 0.05; **p < 0.01; ***p < 0.001. (**A**) Plasama glucose. (**B**) Plasma corticosterone. (**C**) Plasma glucagon.

Figure 6 illustrates VMN GLUT2 gene knockdown effects on plasma glucose and counterregulatory hormone concentrations. Mean circulating glucose levels (Fig. 6A; F_(3,20)_: 266.27, *p* < 0.001, Knockdown main effect: F_(1,44)_: 0.01; *p* = 0.910; INS main effect: F_(1,44)_: 798.78; *p* < 0.001, Knockdown/INS interaction: F_(1,44)_: 0.01; *p* = 0.910) were unaffected by GLUT2 gene silencing. IIH decreased plasma glucose to an equivalent extent in SCR versus GLUT2 siRNA-pretreated animals. As shown in Fig. 6B,C, plasma corticosterone [F_(3,20)_: 51.19, *p* < 0.001, Knockdown main effect: F_(1,44)_: 10.15; *p* = 0.005; INS main effect: F_(1,44)_: 116.65; *p* < 0.001, Knockdown/INS interaction: F_(1,44)_: 26.77; *p* < 0.001] and glucagon profiles [F_(3,20)_: 42.07, *p* < 0.001, Knockdown main effect: F_(1,44)_: 9.63; *p* = 0.006; INS main effect: F_(1,44)_: 115.04; *p* < 0.001, Knockdown/INS interaction: F_(1,44)_: 1.54; *p* = 0.229] in V-injected rats were refractory to prior GLUT2 gene knockdown. However, GLUT2 siRNA pretreatment significantly augmented concentrations of both hormones in INS-injected animals. Data reveal that VMN GLUT2 gene silencing does not affect eu- or hypoglycemic profiles or patterns of corticosterone or glucagon secretion in euglycemic animals. Results however document GLUT2-mediated blunting of the magnitude of these individual hormone profiles in hypoglycemic subjects.

**Figure 7:**
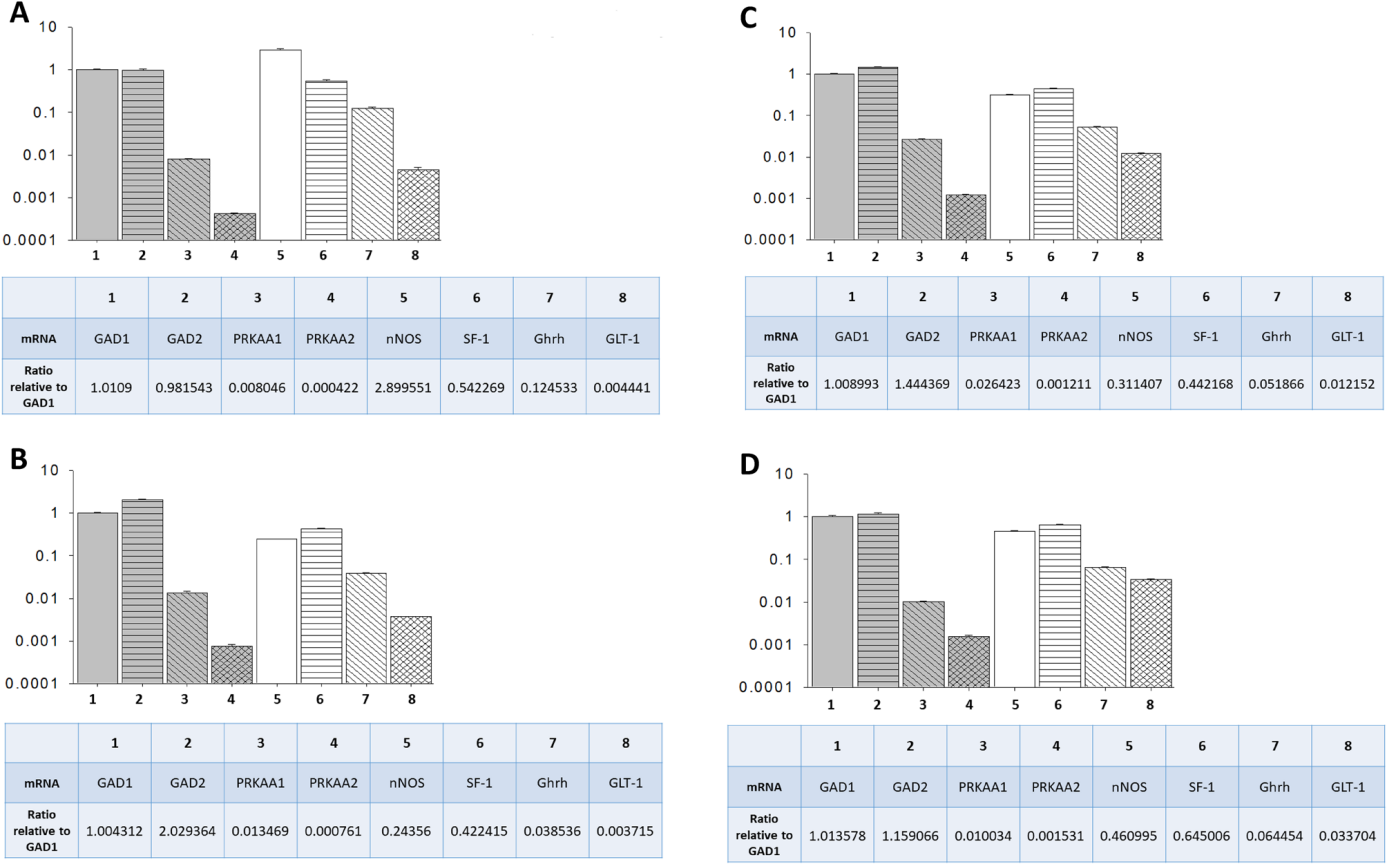
Mean Ratios of Co-Expressed Counterregulatory Transmitter Marker, PRKAA Isoforms, and SF-1 mRNAs Analyzed for VMNdm versus VMNvl GABAergic Neurons. Mean ratios for baseline mRNA values measured by multiplex single-cell qPCR are depicted for laser-catapult-microdissected GAD_65/67_–ir neurons collected from the VMNdm (**A**, *top left*; *n* = 12) versus VMNvl (**B**, *bottom left*; *n* = 12). Mean ratios for hypoglycemic mRNA expression profiles measured by multiplex single-cell qPCR are shown for laser-catapult-microdissected VMNdm (**C**, *top right*; *n* = 12) or VMNvl (**D**, *bottom right*; *n* = 12) GAD_65/67_–ir neurons. (**A**) VMNdm GABA neuron mRNA ratios - Euglycemia. (**B**) VMNvl GABA neuron mRNA ratios - Euglycemia. (**C**) VMNdm GABA neuron mRNA ratios - Hypoglycemia. (**D**) VMNvl GABA neuron mRNA ratios - Hypoglycemia.

Figure 7 illustrates expression ratios of genes of interest, including co-expressed glucose-regulatory neurotransmitter markers, relative to GAD1, for VMNdm (Fig. 7A and C) or VMNvl (Fig. 7B and D) GABA neurons. Data infer that under euglycemic conditions, GABAergic nerve cells acquired from these separate VMN locations exhibit divergent relative ratios of baseline nNOS, Ghrh, and GLT-1 mRNA levels, as proportionate nNOS, Ghrh, and GLT-1 gene expression was greater in the VMNdm versus VMNvl. Comparison of gene expression ratios between eu- versus hypoglycemia animals shows that VMNdm GABA neurons exhibit evident changes in proportionate expression of nNOS, Ghrh, and GLT-1 corresponding to GAD1 transcription, namely reduction in the former two gene ratios, alongside augmentation of the latter. At the same time, hypoglycemia increased relative ratios of expression of nNOS, Ghrh, and GLT-2 in VMNvl GABAergic nerve cells. Notably, baseline ratios of GAD2 versus GAD1 transcript profiles varied between the two divisions, and were affected by hypoglycemia in each site, as this ratio increased in VMNdm neurons, but declined in VMNvl cell samples. Relative gene expression data presented in Fig. 7 show that baseline PRKAA1 transcripts occur at higher levels corresponding to GAD1 compared to PRKAA1 mRNA in GABAergic neurons from each VMN division. Relative PRKAA1 transcription was greater in the VMNvl compared to VMNdm GABA neurons; interestingly, hypoglycemia increased this ratio in the VMNdm, but did not cause evident change in VMNvl neurons. Relative PRKAA2 gene expression was augmented by hypoglycemia in GABA nerve cells from both VMN locations.

## Discussion

The plasma membrane glucose transporter/sensor GLUT2 regulates hypothalamic astrocyte glycolytic pathway function, glycogen metabolism, and energy stability in vitro^48,49^. Here, an in vivo experimental approach was used to investigate whether GLUT2 function in the VMN, a principal element of the brain glucostatic regulatory network, controls VMN GABAergic neuron neurotransmitter signaling and counterregulatory hormone secretion in the female rat. Single-cell multiplex qPCR analyses of laser-catapult-microdissected VMNdm versus VMNvl GAD_65/67_-ir neurons show that these cell subpopulations exhibit dissimilar GLUT2-dependent GAD1 and -2 transcriptional responses to hypoglycemia. Data also provide unique evidence that GABA neurons residing in each VMN division express genes that encode markers for the characterized counterregulatory neurochemicals NO, Ghrh, and glutamate, and show that division-specific NOS1 and GLT-1 mRNA responses to IIH are regulated by GLUT2. Gene expression measurements disclose contrary GLUT2 control of VMNdm versus VMNvl GABAergic neuron SF-1 gene responses to hypoglycemia. Substantiation here that VMN GLUT2 gene silencing enhances hypoglycemic patterns of corticosterone and glucagon secretion infer that sensor input to local glucose-regulatory circuitries is capable of blunting counterregulatory endocrine outflow. Further research is necessary to determine if GLUT2-responsive neurochemical transmission by GABAergic neurons may mediate sensor control of those systemic hormone profiles.

Current research sought to gain proof-of-concept that genetic manipulation of VMN GLUT2 gene expression affects VMNdm and/or VMNvl GABAergic nerve cell energy sensor and co-expressed transmitter marker protein mRNA profiles. Given the considerable quantity of work required to achieve this goal, we chose to use only one sex as experimental subjects here. Present studies were performed using adult female rats as a whole-animal experimental model as previous research in our laboratory investigated effects of trafficable astrocyte metabolic fuel on VMN counterregulatory neurotransmitter nerve cell function in this sex^28,29,31^. The current project is the first to evaluate GAD isoform mRNAs in VMNdm versus VMNvl GABA neurons in female rats. Recent work involving qPCR analysis of GAD1 and GAD2 transcript profiles in GAD_65/67_-ir neurons acquired from the two major subdivisions of the male rat VMN revealed that both GAD mRNAs were down-regulated in the VMNdm and VMNvl in response to IIH^18^. GAD1 and GAD2 enzyme proteins are encoded by different genes, and exhibit dissimilar subcellular distribution (GAD2, but not GAD1 occurs in axon terminals and vesicles) and regulation (GAD1 is governed by transcriptional and posttranscriptional mechanisms; GAD2 is subject to transcriptional and kinetic controls^57^). Differential localization of these enzymes infers that separate cytoplasmic and vesicular GABA pools exist, enabling GAD1 and GAD2 participation in cellular metabolic pathways versus neurotransmission, respectively^58–60^. Present data show that in the female, hypoglycemia up-regulated GAD1 and GAD2 mRNA expression in VMNdm GABA neurons, while inhibiting GAD2 transcription in VMNvl neurons. Comparison of prior and present study outcomes exposes intriguing potential sex differences in GABA cytoplasmic and vesicular accumulation in VMNdm GABAergic neurons collected from hypoglycemic animals, raising the prospect that hypoglycemic patterns of GABA release in that VMN division may be sex-contingent, i.e. increased in female versus decreased in male. This supposition will require experimental verification that measured adjustments in GAD mRNA profiles described here elicit corresponding changes in enzyme protein expression and enzyme activity of similar direction and magnitude. Application here of multiplex qPCR analysis to individual GABAergic neuron cell samples provides a unique opportunity to not only evaluate treatment effects on absolute expression levels of discrete genes of interest, but is also conducive to assessment of potential adjustments in relative transcription patterns. Outcomes shown in Fig. 7 indicate differences in relative baseline expression of GAD2 corresponding to GAD1 between VMNdm versus VMNvl GABAergic neurons, and reveal divergent effects of hypoglycemia on this expression ratio in these divisional populations. These data support the view that GAD isoform-dependent GABA function and effects of hypoglycemia on those functions likely varies between these anatomically-distinct cell groups.

Current results show that in the female, basal VMNdm GABA neuron GAD1 and GAD2 transcripts are respectively suppressed by or are refractory to GLUT2; evidence that GLUT2 stimulates both gene profiles during hypoglycemia points to a shift in direction of GLUT2 regulation of GAD1 gene expression, i.e. from negative to positive, and a gain of control of GAD2 mRNA profiles during eu- versus hypoglycemia. Glucose status is thus a determinant of GLUT2 impact on GAD mRNA profiles in VMNdm GABAergic neurons. Meanwhile, GLUT2 imposes a suppressive tone on hypoglycemic patterns of VMNvl GABA neuron GAD2 gene expression, as GLUT2 gene silencing blunted this inhibitory transcriptional response. Measurement of GLUT2 mRNA in VMNdm versus VMNvl GABA neurons shows that hypoglycemia respectively up- or down-regulates this gene profile in these two subpopulations. Observed parallelism of GLUT2 and GAD2 transcription responses to IIH implies that GABA neurotransmission levels in each VMN division may reflect, in part, summation of individual sensor cues. Hypoglycemia-associated increases or reductions in GABAergic neuron GLUT2 gene expression presumably elicit similar changes in GLUT2 protein production, which in turn would likely affect glucose volume entering those neurons by this specific uptake mechanism. How those supposed changes in GLUT2 mass, e.g. augmented in VMNdm GABA nerve cells while diminished in VMNvl, may affect net glucose internalization during IIH-associated decrements in VMN tissue glucose concentrations [Ibrahim et al., 2020] is not known at present. Concerning the male, the question of whether GLUT2 regulates eu- and/or hypoglycemic VMNdm and VMNvl GABAergic neuron GAD isoform gene expression remains to be addressed. Early studies characterized GABA as inhibitory to counterregulation^61,62^. Continuing research efforts by our group seek to determine if GLUT2-dependent augmentation (VMNdm) or diminution (VMNvl) of this suppressive signal may affect endocrine hormone secretion in the female.

Data presented in Fig. 2 supply unique confirmation that female rat VMNdm and VMNvl GABA neurons express genes that encode the counterregulation-enhancing neurochemicals NO, Ghrh, and glutamate. VMN division-specific NOS1 and Ghrh transcriptional responses to IIH underscore the notion that GABAergic neurons comprise functionally distinct cell populations in these subnuclear sites. Further work is warranted to examine whether these divergent gene responses reflect, in part, contrary effects of hypoglycemia on GLUT2 mRNA in those locations, i.e. up-regulation in VMNdm versus down-regulation in VMNvl. Data show that GLUT2 suppresses baseline NOS1 and GLT-1 gene profiles in both VMN divisions, as well as basal VMNvl Ghrh mRNA content. The direction of GLUT2 regulation of NOS1 and Ghrh gene expression is glucose-dependent, as control is inhibitory during euglycemia, yet stimulatory during hypoglycemia. On the other hand, GLUT2 is apparently a consistent negative stimulus for VMNdm and VMNvl GLT-1 gene transcription regardless of plasma glucose profiles, serving to blunt hypoglycemic up-regulation of this mRNA. Simultaneous transmission of counterregulation-inhibitory and -stimulatory neurotransmitters of differing chemical structure and temporo-spatial release profiles signifies that GABA neurons likely exhibit the capability for complex dynamic input to the glucostatic regulatory circuitry. It would be useful to learn if distinctive neurochemicals communicate unique or redundant information on dynamic aspects of brain cell energy state, metabolic fuel supply, and local/systemic energy reserve capacities. There is also a need for information on how integration of multiple neurochemical signals over different spatial and temporal domains by GABAergic neurons may influence counter-regulation in the female. A related issue requiring resolution involves identification of downstream cellular targets of VMNdm versus VMNvl GABA nerve cell transmitters released by exocytotic versus diffusion mechanisms. Division-based differences in relative gene expression ratios for co-expressed glucose-regulatory transmitters, depicted in Fig. 7, underscore the need to determine to clarify the net effect of VMNdm versus VMNvl GABAergic neuron multi-modal input on neural regulation of glucose homeostasis.

Earlier studies in male rats showed that VMN GABA neurons express the ultra-sensitive energy gauge AMPK, and that its activation state is up-regulated by hypoglycemia^25,26,52^. Here, discriminative analysis of division-based GABAergic neuron populations in the female VMN shows that VMNdm GABA nerve cells exhibit GLUT2-dependent up-regulation of PRKAA1/AMPKα1 and PRKAA2/AMPKα2 gene expression in response to IIH. In contrast, VMNvl GABA neurons show GLUT2-independent augmentation of AMPKα2 transcription, albeit at a lesser-fold increment compared to VMNdm. These data support the notion that each divisional GABA neuron population may exhibit distinctive AMPK responses to IIH. Further research is needed to investigate how hypoglycemia may affect GABA neuron AMPKα1 and AMPKα2 protein expression and, importantly phosphorylation, i.e. activation in each VMN division, and to determine if GLUT2 modulates GABA neuron AMPK activation by IIH. By extension, it will also be useful to learn how AMPK activity state in either GABA cell population shapes transmission of one or more co-expressed neurochemicals, including GABA. Relative gene expression data presented in Fig. 7 infer that in both VMNdm and VMNvl, baseline GABA nerve cell PRKAA1/AMPKα1 transcription, corresponding to GAD1, exceeds that of PRKAA2/AMPKα2. As these catalytic subunit isoforms are activated to a similar extent in response to increases in intracellular AMP, but exhibit dissimilar substrate specificity, discrepant rates of gene transcription likely affect cell function. Hypoglycemia-associated adjustments in relative ratios of AMPK alpha subunit gene expression infer that glucose status is a critical determinant of AMPK regulation of GABA neurons in each VMN location.

Cumulative literature advances the position that the transcription factor SF-1, which is uniquely expressed in the VMN, exhibits a restricted distribution which is limited to the VMNdm. qPCR data reported here indicate that SF-1 mRNA is present in GABA neurons acquired from the VMNdm or VMNvl. Levels of SF-1 protein expression in each GABA nerve cell subpopulation remain to be quantified. Data here show that GLUT2 inhibits baseline SF-1 gene transcription in VMNvl GABA neurons, but not in the VMNdm population. GLUT2 was found to impose opposite control of hypoglycemic patterns of SF-1 mRNA expression in VMNdm versus VMNvl GABA neurons, as results indicate that it drives (VMNdm) or curbs (VMNvl) this positive transcriptional response. It remains to be determined if SF-1 may mediate GLUT2 regulatory effects on neurotransmitter gene expression in each GABA cell population.

Use here of in vivo GLUT2 gene silencing techniques produced novel evidence that VMN GLUT2 function shapes systemic counterregulatory hormone profiles in the hypoglycemic female rat. Results reveal that in this sex, VMN GLUT2 imposes a net negative influence on hypercorticosteronemia and hyperglucagonemia, acting as a physiological brake to these stimulatory endocrine responses. Data disclose that the current gene knockdown paradigm did not modify plasma glucose concentrations in INS-injected animals despite augmenting counterregulatory hormone output. Since glucose measurement was limited to time of sacrifice, the prospect that hypoglycemic profiles were affected by VMN GLUT2 gene knockdown at one or more time points prior to + 1 h should not be dismissed. It is possible that plasma glucose levels may exhibit dynamic change as a consequence of VMN GLUT2 control of the counterregulatory hormones investigated here and/or adrenomedullary regulation of hepatic gluconeogenic or glycogenolytic functions. Outcomes described here may represent a temporal phase during which INS-induced decrements in circulating glucose are normalized in response to preceding GLUT2-dependent adjustments in counterregulatory hormone secretion. In light of current evidence for distinctive GLUT2 regulation of divisional GABA nerve cell populations, it is intriguing to speculate whether GLUT2 action within the VMNdm versus VMNvl may have divergent effects on these endocrine profiles. Further research is required to investigate whether GABA neurons in one or both divisions serve as the sole conduit of GLUT2 regulatory cues to the brain glucostatic circuitry, or if this sensor may also control the functional status of other VMN neurotransmitter nerve cell types that operate within that network. It also remains to be determined if and how integration of GABA and co-expressed neurochemical signals affect hypoglycemic pattern of corticosterone and glucagon secretion. It is imperative that future research identify the neuroanatomical location(s) and cellular targets of GLUT2-dependent VMN GABAergic neurochemical signaling within the neural glucose-regulatory circuitry, including the prospect that this nerve cell population may directly or indirectly influence neurotransmitter function within other hypothalamic metabolic loci, namely the arcuate and dorsomedial hypothalamic nuclei and lateral hypothalamic area.

Based upon existing reports that GLUT2 functions within astrocytes to govern counterregulation^17^, the working premise of current research was that manipulation of VMN GLUT2 expression will likely affect astrocyte metabolic coupling with local neurons, including GABA nerve cells, thereby impacting glucose-regulatory signaling by that neuron population. Validation of this supposition will require application, in future research, of approaches that achieve brain cell type-specific GLUT2 gene silencing^63^. The alternative prospect that GLUT2 imposes direct control of VMNdm and/or VMNvl GABAergic neuron energy sensor and transmitter marker mRNA profiles is not supported by current literature. However, this scenario should not be not discounted in the absence of definitive evidence that this VMN nerve cell type either expresses or does not express GLUT2.

It is notable that the magnitude of reductions in astrocyte GLUT2 mRNA levels subsequent to intra-VMN GLUT2 siRNA administration varied between the VMNdm and VMNvl. Current research does not identify potential factors that may be responsible for this discrepancy, but it is speculated that dissimilar treatment-associated GLUT2 mRNA down-regulation may reflect either differences in amount of siRNA reaching astrocytes located in the VMNdm versus VMNvl and/or dissimilar astrocyte responses to a given siRNA dosage. The selection of the central VMN, located between the VMNdm and VMNvl, as the target site for siRNA delivery supports the likelihood that equivalent amounts of siRNA were accessible by diffusion to astrocytes in those locations. With regard to the latter scenario, it is intriguing to consider that astrocytes may exhibit dissimilar adaptive responses to down-regulated GLUT2 mRNA levels in the VMNdm versus VMNvl, such as differential adjustments in metabolism of remaining GLUT2 transcripts.

In summary, current research utilized in vivo investigative strategies to address the novel concept that in the female, VMN GLUT2 shapes counterregulatory endocrine responses to hypoglycemia, and GABAergic neurons located in one or both subnuclear sites examined here may be an effector of that control. There is substantial existing evidence that implicates GABAergic neurotransmission in hypothalamic control of counterregulatory hormone secretion, including studies by Sherwin and his colleagues. Present data that describe VMN division-specific effects of GLUT2 gene silencing on hypoglycemic patterns of GABA nerve cell GAD1 and GAD2 gene transcription support the notion that one or both of these distinctive GABA neuron populations may be a potential effector of GLUT2 glucose sensory input to the brain glucostatic regulatory network. Results infer that VMN GABA nerve cells may engage in multi-modal neurochemical signaling, as data uniquely document co-expression of counterregulation-inhibitory and –stimulatory neurochemicals of varying chemical structure and spatial/temporal release characteristics in these cells. This complexity of neurotransmission may permit integrated input of diverse types of relevant metabolic information to the brain glucostatic network. Ongoing research seeks to determine if GLUT2 regulatory signals affect GABA neuron neurotransmission by AMPK- and/or SF-1-dependent mechanisms. Further effort is also required to characterize the mechanism(s) of communication by which astrocytes convey GLUT2 cues to VMN neurons that contribute to neural regulation of glucose homeostasis.

## Data Availability

The data that support the findings of this study are available from the corresponding author upon reasonable request.

## Abbreviations

AMPKα1: 5’-AMP-activated protein kinase-alpha1
AMPKα2: 5’-AMP-activated protein kinase-alpha2
GAD1/GAD_67_: Glutamate decarboxylase_67_
GAD2/GAD_65_: Glutamate decarboxylase_65_
Ghrh: Growth hormone-releasing hormone
GLUT2: Glucose transporter-2
IIH: Insulin-induced hypoglycemia
INS: Insulin
NOS1/nNOS: Neuronal nitric oxide synthase
NO: Nitric oxide
OVX: Ovariectomy
*Sc*: Subcutaneous
SF-1/Nr5a1: Steroidogenic factor-1
Slc1A2/GLT-1: Vesicular glutamate transporter-1
